# Plasma glial fibrillary acidic protein and tau: predictors of neurological outcome after cardiac arrest

**DOI:** 10.1186/s13054-024-04889-0

**Published:** 2024-04-09

**Authors:** Isabelle Arctaedius, Helena Levin, Bergthóra Thorgeirsdóttir, Marion Moseby-Knappe, Tobias Cronberg, Martin Annborn, Niklas Nielsen, Henrik Zetterberg, Kaj Blennow, Nicholas J. Ashton, Attila Frigyesi, Hans Friberg, Anna Lybeck, Niklas Mattsson-Carlgren

**Affiliations:** 1 Department of Clinical Sciences, Anaesthesia and Intensive Care, Skane University Hospital, Lund University, Lund, Sweden; 2 Department of Research and Education, Skane University Hospital and Department of Clinical Sciences, Anaesthesia and Intensive Care, Lund University, Lund, Sweden; 3 Department of Clinical Sciences, Anaesthesia and Intensive Care, Skane University Hospital, Lund University, Malmö, Sweden; 4 Neurology and Rehabilitation Medicine, Department of Clinical Sciences Lund, Skane University Hospital, Lund University, Lund, Sweden; 5 Department of Clinical Sciences, Neurology, Skane University Hospital, Lund University, Lund, Sweden; 6 Department of Clinical Sciences, Anaesthesia and Intensive Care, Helsingborg Hospital, Lund University, Helsingborg, Sweden; 7 Clinical Neurochemistry Laboratory, Sahlgrenska University Hospital, Mölndal, Sweden; 8 Department of Psychiatry and Neurochemistry, Institute of Neuroscience and Physiology, The Sahlgrenska Academy at the University of Gothenburg, Mölndal, Sweden; 9 Department of Neurodegenerative Disease, UCL Institute of Neurology, Queen Square, London, UK; 10 UK Dementia Research Institute at UCL, London, UK; 11 Hong Kong Center for Neurodegenerative Diseases, Clear Water Bay, Hong Kong China; 12 Wisconsin Alzheimer’s Disease Research Centre, University of Wisconsin School of Medicine and Public Health, University of Wisconsin-Madison, Madison, WI USA; 13 Institute of Psychiatry, Psychology and Neuroscience, King’s College London, London, UK; 14 NIHR Biomedical Research Centre for Mental Health and Biomedical Research Unit for Dementia at South London and Maudsley NHS Foundation, London, UK; 15 Centre for Age-Related Medicine, Stavanger University Hospital, Stavanger, Norway; 16 Clinical Memory Research Unit, Department of Clinical Sciences, Lund University, Malmö, Sweden; 17 Wallenberg Centre for Molecular Medicine, Lund University, Lund, Sweden

**Keywords:** Heart arrest, In-hospital cardiac arrest, Out-of-hospital cardiac arrest, Prognostication, Biomarkers, Tau, GFAP

## Abstract

**Background:**

The purpose was to evaluate glial fibrillary acidic protein (GFAP) and total-tau in plasma as predictors of poor neurological outcome after out-of-hospital (OHCA) and in-hospital cardiac arrest (IHCA), including comparisons with neurofilament light (NFL) and neuron-specific enolase (NSE).

**Methods:**

Retrospective multicentre observational study of patients admitted to an intensive care unit (ICU) in three hospitals in Sweden 2014–2018. Blood samples were collected at ICU admission, 12 h, and 48 h post-cardiac arrest. Poor neurological outcome was defined as Cerebral Performance Category 3–5 at 2–6 months after cardiac arrest. Plasma samples were retrospectively analysed for GFAP, tau, and NFL. Serum NSE was analysed in clinical care. Prognostic performances were tested with the area under the receiver operating characteristics curve (AUC).

**Results:**

Of the 428 included patients, 328 were OHCA, and 100 were IHCA. At ICU admission, 12 h and 48 h post-cardiac arrest, GFAP predicted neurological outcome after OHCA with AUC (95% CI) 0.76 (0.70–0.82), 0.86 (0.81–0.90) and 0.91 (0.87–0.96), and after IHCA with AUC (95% CI) 0.77 (0.66–0.87), 0.83 (0.74–0.92) and 0.83 (0.71–0.95). At the same time points, tau predicted outcome after OHCA with AUC (95% CI) 0.72 (0.66–0.79), 0.75 (0.69–0.81), and 0.93 (0.89–0.96) and after IHCA with AUC (95% CI) 0.61 (0.49–0.74), 0.68 (0.56–0.79), and 0.77 (0.65–0.90). Adding the change in biomarker levels between time points did not improve predictive accuracy compared to the last time point. In a subset of patients, GFAP at 12 h and 48 h, as well as tau at 48 h, offered similar predictive value as NSE at 48 h (the earliest time point NSE is recommended in guidelines) after both OHCA and IHCA. The predictive performance of NFL was similar or superior to GFAP and tau at all time points after OHCA and IHCA.

**Conclusion:**

GFAP and tau are promising biomarkers for neuroprognostication, with the highest predictive performance at 48 h after OHCA, but not superior to NFL. The predictive ability of GFAP may be sufficiently high for clinical use at 12 h after cardiac arrest.

**Supplementary Information:**

The online version contains supplementary material available at 10.1186/s13054-024-04889-0.

## Background

Among patients admitted to intensive care after cardiac arrest, the most common cause of death is related to brain injury, preceded by withdrawal of life-sustaining therapies (WLST) after the prediction of a poor neurological outcome [1]. The 2021 ERC/ESICM guidelines recommend multimodal neuroprognostication from 72 h after a cardiac arrest based on a combination of clinical examination, biomarkers of brain injury, neurophysiological investigations, and neuroimaging [2]. The majority of studies on post-cardiac arrest care, including neuroprognostication and biomarkers of brain injury, have been performed after out-of-hospital cardiac arrest (OHCA), with results incorporated into guidelines also informing care after in-hospital cardiac arrest (IHCA) [2]. Biomarkers in the blood are suitable tools for prognostication since they are easily obtained, results are quantitative and objective, and they are not known to be affected by sedatives or muscle relaxants. The current ERC/ESICM guidelines recommend one biomarker of brain injury—neuron-specific enolase (NSE), with a predictive value 48-72 h after cardiac arrest [2]. Novel biomarkers, including neurofilament light (NFL), glial fibrillary acidic protein (GFAP), total-tau, and phosphorylated tau, have shown promising prognostic value after cardiac arrest [3–12]. GFAP is an intermediate-filament component in the astrocyte cytoskeleton and is released from damaged or activated astrocytes [3, 13]. GFAP is elevated in serum or plasma after cardiac arrest, intracerebral haemorrhage, head trauma, and ischemic stroke [14–18]. Elevated GFAP may also be seen in healthy individuals with developing Alzheimer's pathology, but levels are lower than those seen after cardiac arrest [19]. Tau protein is a microtubule-stabilising structure found in smaller unmyelinated axons and is released non-specifically after brain injury. Elevated tau concentrations in serum or plasma have been described after ischemic stroke and cardiac arrest [3, 4, 13]. In selected groups of comatose survivors after OHCA, tau has an excellent ability (AUC 0.91–0.93) to predict poor outcomes at 48 h. [4, 5]. The predictive ability of GFAP has differed between studies [4, 7–9, 11, 20, 21]. Excellent prediction (AUC 0.83–0.91) between 24-72 h post-cardiac arrest has been reported in selected OHCA, whereas AUC as low as 0.59–0.67 were reported in a small study on OHCA and a small mixed sample of OHCA and IHCA.

This study aimed to evaluate the predictive ability of GFAP and tau to predict neurological outcome in OHCA and IHCA at multiple time points. We also compared the predictive ability of GFAP and tau to NSE, the only biomarker of post-hypoxic brain injury used in clinical practice, and to NFL (which has shown promising predictive abilities). Additionally, we wanted to test if a combination of biomarkers originating from astroglial cells and neurons improved prediction compared to individual biomarkers. We, therefore, tested a combination of GFAP and tau and a combination of GFAP and NFL, considering previously described very high predictive values of NFL at 12 h in the same patients [22].

## Methods

### Study setting

Retrospective multicentre observational study of post-cardiac arrest patients admitted to three intensive care units (ICU) in Skåne, Sweden, between 2014–2018. The study was a part of the SWECRIT biobank project (https://clinicaltrials.gov/ct2/show/NCT04974775), which aimed to include all adults admitted to the involved ICUs to study biomarkers in critically ill patients [22]. The Standards for Reporting Diagnostic Accuracy Studies (STARD) guidelines were followed [23].

### Study participants and outcomes

Blood samples were collected at ICU admission and 12 h and 48 h post-cardiac arrest. Cardiac arrest patients were identified by ICD-10 in the local intensive care registry. Patients with only an admission sample (drawn < 6 h after cardiac arrest) were excluded. Samples drawn within six hours of the specified time points were included for statistical analysis. Patient data were collected from medical records, the International Cardiac Arrest Registry (INTCAR), the local ICU registry, and the Swedish population register. During the study period, post-cardiac arrest care was given according to current guidelines, including target temperature management (TTM) at 36 °C for 28 h and multimodal neuroprognostication at ≥ 72 h for all unconscious patients. Withdrawal of life-sustaining therapies (WLST) was practised, and information on time to WLST and reasons for WLST (neurological, circulatory failure/multi-organ failure, comorbidity, or ethical reasons) were collected [24].

Long-term neurological outcome was assessed by any healthcare professional at 2–6 months after cardiac arrest according to the Cerebral Performance Category (CPC) scale. Good outcome was defined as CPC 1–2 (good cerebral performance or moderate cerebral disability), and poor outcome as CPC 3–5 (severe cerebral disability, coma, or brain death).

### Biomarker analysis

The local laboratory centrifuged, aliquoted, and froze plasma samples at each site before they were sent to the biobank at Region Skåne, Sweden (BD-47, SC-1922) for long-term storage until analysis. GFAP, total-tau, and NFL levels were analysed using commercially available Single molecule array (Simoa) assays on an HD-X instrument (Quanterix, Billerica, MA) at the Clinical Neurochemistry Laboratory at the University of Gothenburg by staff blinded to all clinical data. NSE was prospectively analysed 24 h and 48 h after cardiac arrest, using an electrochemiluminescence immunoassay on cobas e601/e602 instruments (Roche Diagnostics, Mannheim, Germany). NSE levels were available to treating physicians and employed in multimodal neuroprognostication in clinical practice. NSE levels were not reported for samples with significant haemolysis [25].

### Statistics

Patient characteristics are presented as median and interquartile range (IQR) for continuous variables and as counts and percentages for categorical variables. Categorical variables were compared using the Chi-squared test and continuous variables with the Mann–Whitney U test or logistic regression. The diagnostic performance of the biomarkers was assessed by calculating the area under the receiver operating characteristic curve (AUC) from the logistic regression models. The DeLong method was used to compare AUCs. We examined if the change in GFAP and tau levels between time points (delta) improved the performance compared to individual time points using logistic regression and AUC. We determined the cut-off values of GFAP and tau for poor prognosis at low false-positive rates (FPR) and good prognosis at low false-negative rates (FNR) based on the receiver operating characteristic curve (ROC) coordinate points. To reduce the skewness of the biomarker measurements, we used log10-transformed data. The false discovery rate (FDR) was used to adjust *p*-values for multiplicity. Significance was set at *p* < 0.05. Statistical analyses were performed with R, version 4.1.2 (The R Foundation for Statistical Computing).

### Ethics and consent

The Regional Ethical Review Board in Lund, Sweden, approved the study protocol (registration no. 2014/47 and 2022-02681-01). For patients who regained consciousness, written informed consent was obtained.

## Results

**Fig. 1:**
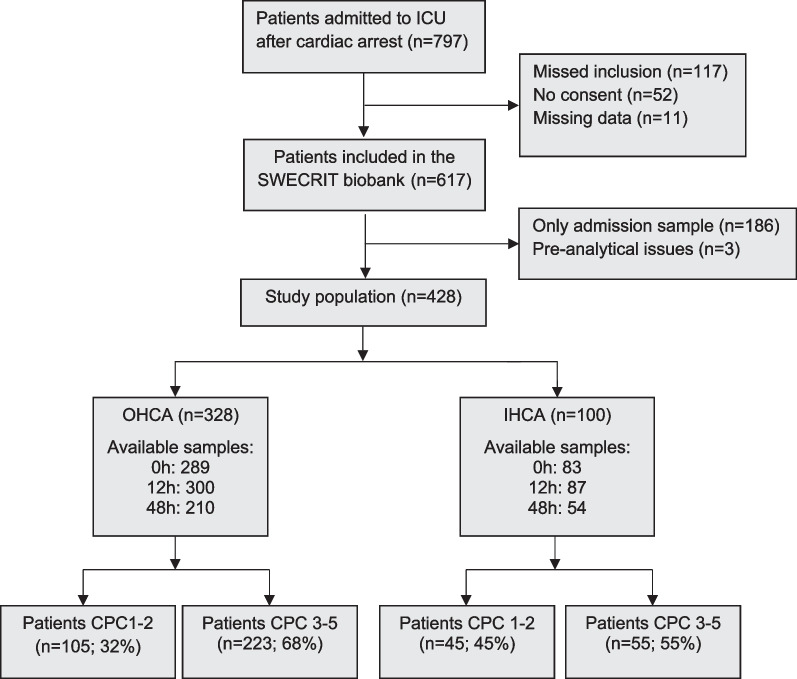
Study flowchart. Long‑term outcomes, according to the Cerebral Performance Category (CPC) scale, were dichotomised into good (CPC 1–2) and poor (CPC 3–5) outcomes. *ICU* intensive care unit, *OHCA* out‑of‑hospital cardiac arrest, *IHCA* in‑hospital cardiac arrest

Four hundred twenty-eight patients were included, 328 OHCA and 100 IHCA (Fig. 1). Characteristics of included compared to missed and excluded patients have previously been published for the cohort used in this study [22]. Cardiac arrest characteristics differed between OHCA and IHCA (Table 1). In OHCA, the time to ROSC was longer, a shockable first recorded rhythm and a cardiac cause were more common; patients who suffered IHCA had a higher Glasgow Coma Scale Motor Score (GCS-M) on admission, a good neurological outcome was more common, and IHCA were less often subject to WLST, particularly WLST due to poor neurological prognosis (Additional file 1: eTable 1). After sample collection, plasma samples were frozen within a median of 1.25 h (IQR 1.0–2.1 h).

**Table 1:** Patient characteristics

	OHCA (n = 328)	IHCA (n = 100)	*p*-value
Age, year—median	67 [59–75]	71 [59–77]	0.09
Sex, Male	247 (75.3)	63 (63.0)	0.02
**Medical history**			
Myocardial infarction	50 (15.2)	19 (19.0)	0.46
Congestive heart failure	52 (15.9)	26 (26.0)	0.03
Hypertension	124 (37.8)	48 (48.0)	0.09
Liver disease	6 (1.8)	4 (4.0)	0.40
Renal disease	24 (7.3)	18 (18.0)	0.003
Diabetes	66 (20.1)	37 (37.0)	0.001
Cerebrovascular disease	25 (7.6)	15 (15.0)	0.04
Dementia/cognitive impairment	13 (4.0)	6 (6.0)	0.56
Solid tumour	31 (9.5)	17 (17.0)	0.06
**Cardiac arrest variables**			
Minutes to ROSC—median	25 [15–40]	10 [6–20]	< 0.001
Witnessed cardiac arrest	256 (78.0)	83 (83.0)	0.35
Bystander CPR	206 (62.8)	N/A	N/A
Arrest with medical personnel present	39 (11.9)	100 (100.0)	N/A
Shockable rhythm	177 (54.1)^c^	21 (21.2)^c^	< 0.001
Cardiac cause^a^	246 (75.0)	38 (38.0)	< 0.001
**Admission data**			
GCS-M—median	1 [1, 2]^g^	3 [1–5]^e^	< 0.001
Circulatory shock	103 (31.4)	32 (32.0)	1.00
**ICU data**			
Patients regaining consciousness	128 (39.3)^d^	66 (66)	< 0.001
Days from CA to awakening	1 [1, 2]^e^	1 [0–2]	0.001
WLST^b^	181 (55.2)	34 (34.0)^c^	< 0.001
WLST due to poor neurological prognosis	159 (48)^f^	26 (26)^d^	< 0.001
Days from CA to WLST	4 [2–5]^f^	4 [3–6]^d^	0.15
**Follow-up**			
Good outcome, CPC 1–2	105 (32.0)	45 (45.0)	0.02
Poor outcome, CPC 3–5	223 (68.0)	55 (55.0)	0.02

Continuous variables are presented as median [interquartile range] and categorical variables as n (%). Proportions (%) are within the groups of OHCA and IHCA patients

^a^Retrospectively diagnosed during the hospital stay

^b^There can be multiple reasons for WLST, including neurological, circulatory, multi-organ failure, comorbidities, or ethical reasons

Missing data: ^c^n = 1, ^d^n = 2, ^e^n = 3, ^f^n = 4, ^g^n = 5

*ROSC* return of spontaneous circulation, *GCS-M* Glasgow Coma Scale Motor response, *ICU* intensive care unit, *CPC* Cerebral Performance Category, *WLST* withdrawal of life-sustaining therapy, *CA* cardiac arrest *OHCA* out-of-hospital cardiac arrest, *IHCA* in-hospital cardiac arrest

### Predictive performance of GFAP

**Fig. 2:**
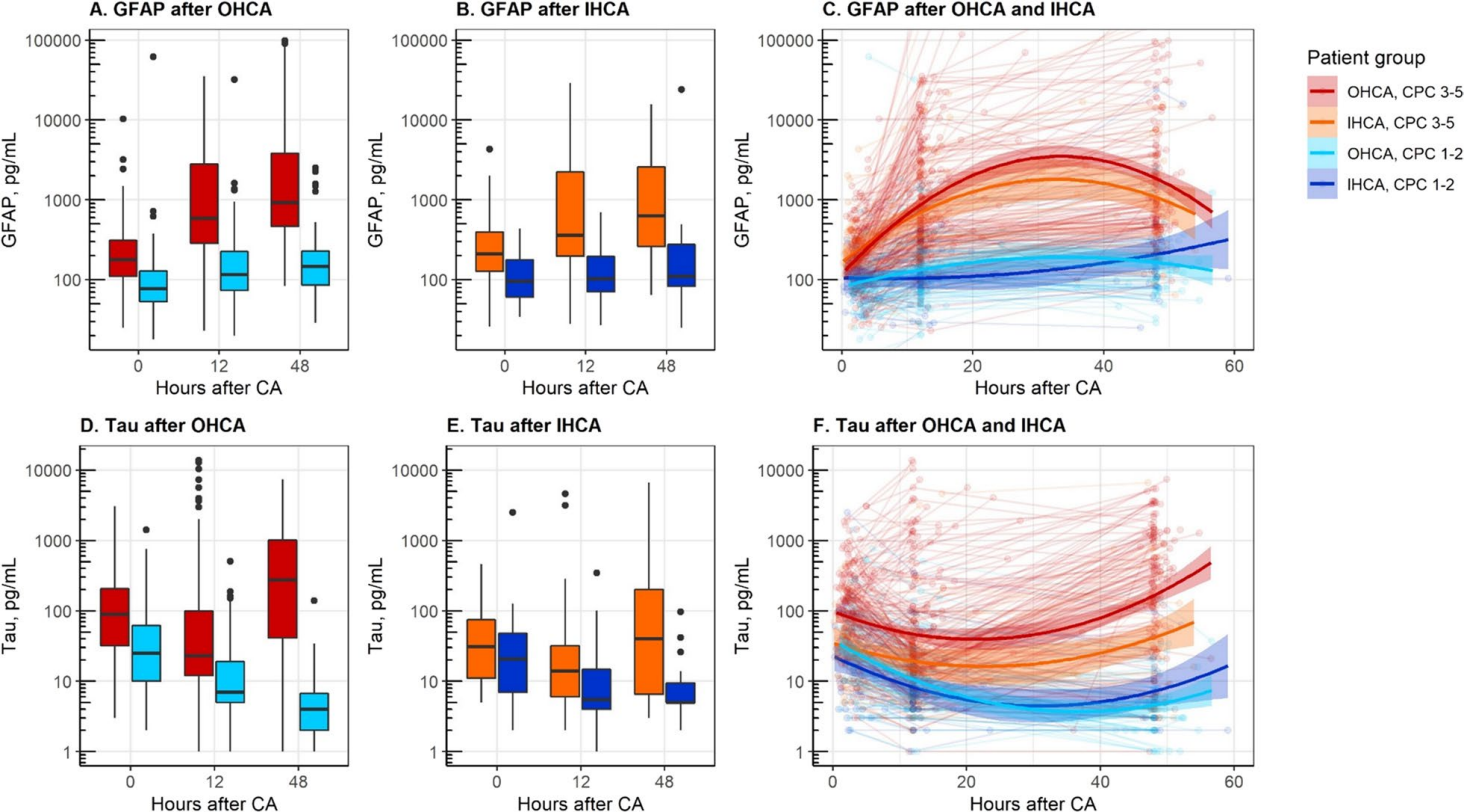
**A**–**F** Plasma GFAP and tau by time point, cardiac arrest group, and outcome. Boxplots are shown for GFAP in panels **A**, **B** and tau levels in panels **D**, **E** at ICU admission, 12 and 48 h post cardiac arrest for OHCA and IHCA, respectively. The boxes show the median and interquartile range (IQR). The bold lines in panels **C** and **F** show the trajectory trend, smoothed via generalised additive models, of GFAP and tau with 95% CI. The dots represent samples and the lines patients. Additional samples (n = 60) collected outside the defined time points (admission [0–6 h], 12 ± 6 h, and 48 ± 6 h post-arrest) were included. *GFAP* glial fibrillary protein, *OHCA* out‑of‑hospital cardiac arrest, *IHCA* in‑hospital cardiac arrest, *CPC* Cerebral Performance Category

**Fig. 3:**
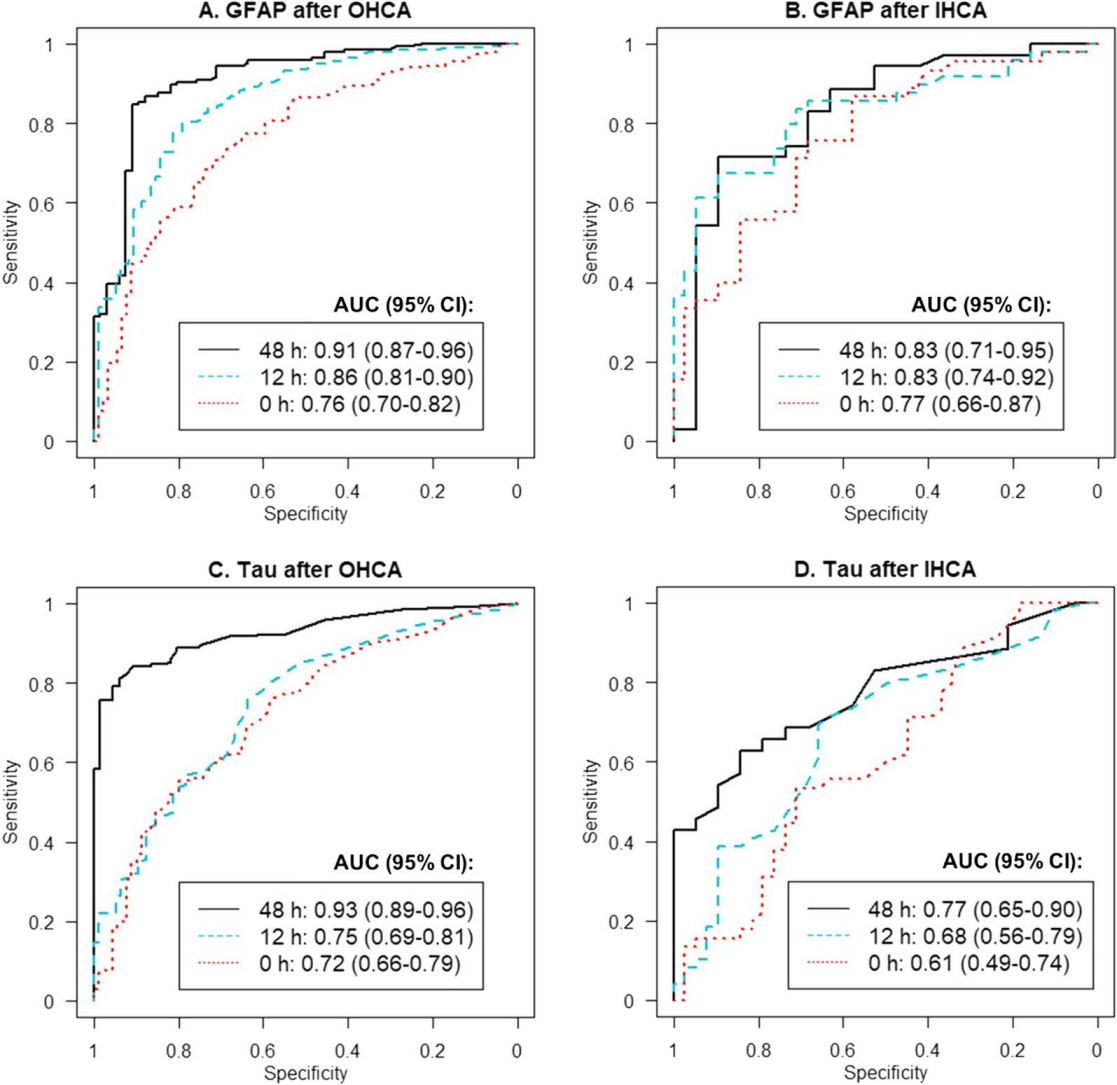
**A**–**D** Prediction of poor vs good outcome using plasma GFAP and tau. The area under the receiver operating characteristic curve (AUC), with 95% confidence intervals, is shown for GFAP and tau at ICU admission, 12 and 48 h after OHCA and IHCA. *GFAP* glial fibrillary protein, *OHCA* out‑of‑hospital cardiac arrest, *IHCA* in‑hospital cardiac arrest

GFAP levels are shown in Fig. 2A, B at ICU admission, 12 h, and 48 h, and Fig. 2C for all available data. GFAP levels were higher in patients with poor outcome compared to patients with good outcome at all pre-defined time points in OHCA (*p* < 0.001) and in IHCA (*p* < 0.003) (Fig. 2A, B). The predictive accuracy expressed as ROC curves is shown in Fig. 3A, B. At admission after OHCA, GFAP had an AUC of 0.76 (95% CI 0.70–0.82), at 12 h, an AUC of 0.86 (95% CI 0.81–0.90), and at 48 h, the AUC was 0.91 (95% CI 0.87–0.96). After IHCA predictive performance was AUC 0.77 (95% CI 0.66–0.87) at admission, AUC 0.83 (95% CI 0.74–0.92) at 12 h and 0.83 (95% CI 0.71–0.95) at 48 h post-cardiac arrest. The prognostic performance remained similar after excluding patients who obeyed commands (GCS-M 6) on admission (Additional file 1: eTable 2). The cause of arrest (cardiac versus non-cardiac) did not significantly affect the predictive ability of GFAP in OHCA or IHCA (Additional file 1: eTable 3 and eFigure 1). The cut-off for prediction of poor outcome with FPR ≤ 2% after OHCA was at 12 h 1626 pg/mL with sensitivity 0.34 (95% CI 0.27–0.41), and at 48 h 2376 pg/ml with sensitivity 0.32 (95% CI 0.24–0.40) (Additional file 1: eTable 4). The cut-off for prediction of good outcome with FNR ≤ 5% was after OHCA at 12 h 111 pg/mL with specificity 0.47 (95% CI (0.38–0.56), and at 48 h 183 pg/ml with specificity 0.64 (95% CI (0.52–0.76) (Additional file 1: eTable 5).

### Predictive performance of tau

Tau levels at ICU admission, 12 h, and 48 h are shown in Fig. 2D–E and in Fig. 2F for all available longitudinal data. Tau levels were higher in patients with poor outcome compared to patients with good outcome at admission, 12 h and 48 h after OHCA (*p* < 0.001) and at all time points after IHCA (*p* < 0.02) except at admission (*p* = 0.08) (Fig. 2D, E). In all patients, irrespective of outcome, tau levels had a pattern with an initial release following cardiac arrest with a subsequent decrease (Fig. 2F). In patients with poor outcome, tau levels rose again toward 48 h (Fig. 2F). The predictive ability of tau was highest at 48 h post-cardiac arrest after both OHCA (AUC 0.93 (95% CI 0.89–0.96)) and IHCA (AUC 0.77 (95% CI 0.65–0.90)) (Fig. 3C, D). The predictive ability was similar after excluding patients with GCS-M 6 on admission (Additional file 1: eTable 2). The cause of cardiac arrest (cardiac versus non-cardiac) did not significantly affect the prognostic ability of tau in OHCA or IHCA (Additional file 1: eTable 3 and eFigure 1). At 48 h after OHCA, the cut-off for prediction of poor outcome with FPR ≤ 2% was 35.5 pg/ml with sensitivity 0.76 (95% CI 0.69–0.83) (Additional file 1: eTable 6) and for prediction of good outcome with FNR ≤ 5% 3.5 pg/ml with specificity 0.45 (95% CI (0.33–0.58) (Additional file 1: eTable 7).

### Serial measures of GFAP and tau

For each biomarker, we tested if adding the change of levels between time points (delta) improved the predictive accuracy compared to using only the last time point. At no time point did the delta improve the predictive accuracies compared to the last single time point (Additional file 1: eTable 8 and 9).

### Plasma GFAP versus tau

The predictive ability of GFAP versus tau and the combination of GFAP and tau versus either biomarker alone is presented in Additional file 1: eTable 10. GFAP was superior to tau at 12 h after both OHCA (*p* = 0.003) and IHCA (*p* = 0.04), and its predictive value was not improved by adding tau at any time after OHCA or IHCA. The predictive value of tau was improved by the addition of GFAP at admission and 12 h after both OHCA (*p* = 0.02, *p* < 0.001) and IHCA (*p* = 0.04, *p* = 0.03) but not at 48 h.

### Plasma GFAP versus NSE

**Fig. 4:**
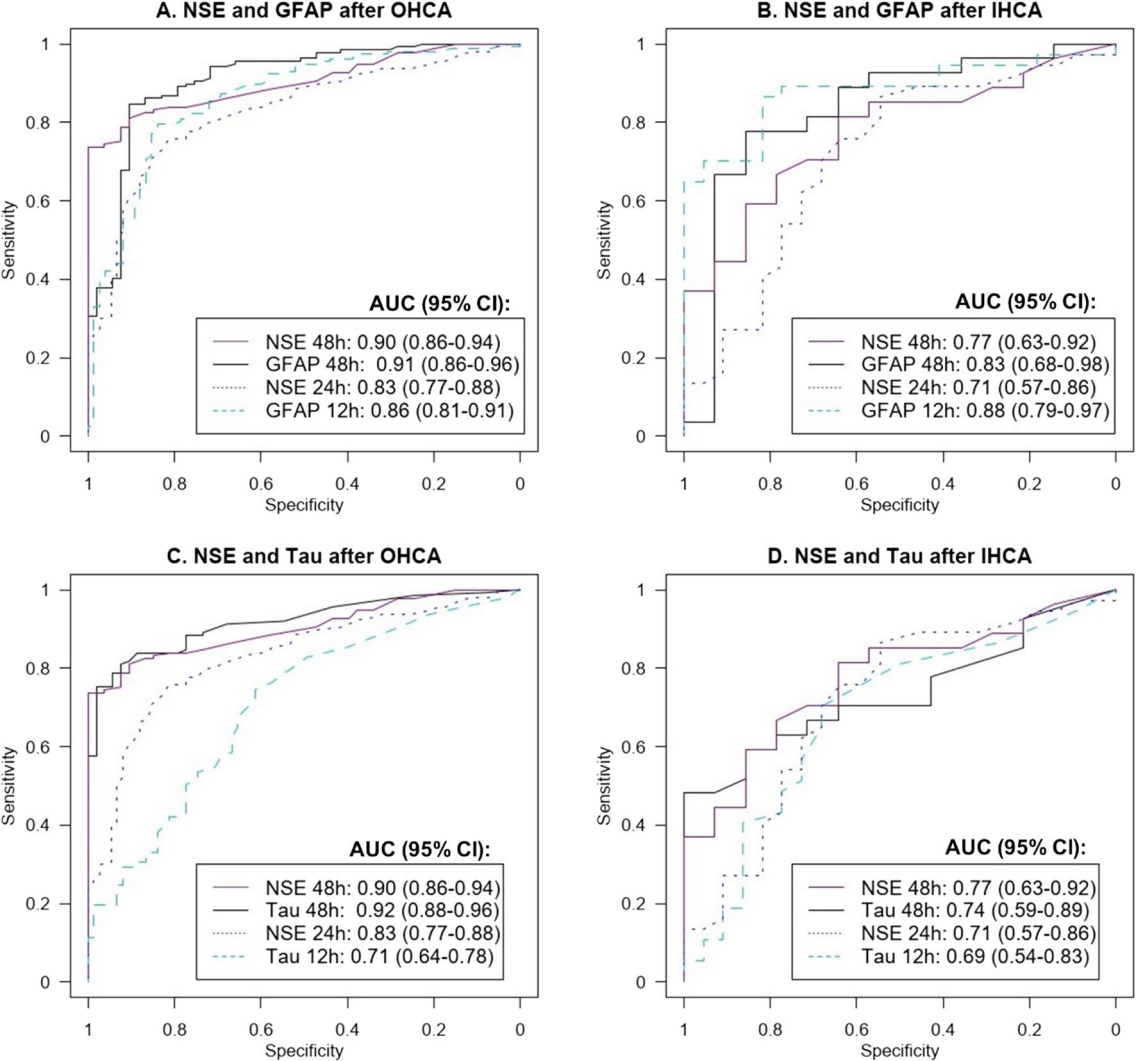
**A**–**D** Classification of poor versus good outcome using NSE, GFAP, and tau. Receiver operatic characteristic curves (ROC) and the area under the curve (AUC), with 95% confidence intervals, are shown in a subgroup of patients with NSE levels at 48 h and 24 h post arrest and GFAP or tau at 48 h and 12 h, respectively. Only participants with both NSE and GFAP or tau levels were included (at 48 h; 190 OHCA and 41 IHCA, at 12/24 h; 232 OHCA and 59 IHCA), and therefore, the results for GFAP and tau differ slightly from those in Fig. 3. *NSE* neuron-specific enolase, *GFAP* glial fibrillary protein, *OHCA* out‑of‑hospital cardiac arrest, *IHCA* in‑hospital cardiac arrest

The predictive abilities of GFAP and NSE are shown in Fig. 4A, B and Additional file 1: eTable 11. Comparisons between NSE and GFAP were made in subsets of patients with available biomarker levels at the compared time points. After both OHCA and IHCA, the predictive ability of GFAP at 12 h was similar to NSE at 48 h after both OHCA (*p* = 0.08) and IHCA (*p* = 0.08). At 48 h, the predictive values of GFAP and NSE were similar after OHCA (*p* = 0.86) and IHCA (*p* = 0.53).

### Plasma tau versus NSE

The predictive abilities of tau and NSE in patients with available NSE levels are shown in Fig. 4C, D and Additional file 1: eTable 12. At 48 h, tau and NSE had similar predictive values after OHCA (*p* = 0.59) and IHCA (*p* = 0.62).

### Plasma GFAP versus NFL

GFAP and NFL had a similar predictive performance at all time points after IHCA and admission after OHCA (Additional file 1: eTable 13). After OHCA, NFL was superior to GFAP at 12 h and 48 h (*p* = 0.01, *p* = 0.02). Compared to GFAP alone, adding NFL to GFAP improved predictive accuracy at 12 h and 48 h after OHCA (*p* < 0.001, *p* = 0.005) but not at admission or after IHCA.

### Plasma tau versus NFL

Tau and NFL had a similar predictive performance at all time points after IHCA and admission after OHCA (Additional file 1: eTable 14). NFL was superior to tau at 12 h and 48 h after OHCA (*p* < 0.001, *p* = 0.004). Compared to tau alone, adding NFL to tau improved the predictive accuracy at all time points after OHCA (*p* = 0.004, *p* < 0.001, *p* < 0.003) and 12 h after IHCA (*p* = 0.04).

## Discussion

In this multicentre observational study, we found that GFAP and tau are reliable predictors of neurological outcome at 48 h after OHCA. Additionally, GFAP displayed excellent performance already 12 h post-cardiac arrest, regardless of the location (OHCA or IHCA) or cause of the arrest (cardiac vs non-cardiac), but was not a reliable predictor of outcome at ICU admission. The predictive value of tau was low at 12 h after cardiac arrest and at ICU admission.

The predictive performance of GFAP at 48 h after OHCA in our study was similar to that found in other studies at 24 h and 48 h after OHCA, including one cohort where WLST was not practised [4, 7–9]. Forty-eight hours after IHCA, GFAP showed considerably better predictive performance than earlier reported in a small mixed population of OHCA and IHCA, possibly explained by case mix, different laboratory methods, or sample size [21]. The major novel finding of our study is the high predictive value of GFAP already at 12 h after both OHCA and IHCA. The release pattern of GFAP into the blood after cardiac arrest included an early and continuous rise in patients with poor neurological outcomes, likely explaining the predictive value at 12 h. In addition, low GFAP levels reliably predicted good neurological outcomes. The predictive value of GFAP at admission was not clinically useful, as observed in previous studies [4, 21].

The only biomarker recommended by guidelines is NSE from 48 h after cardiac arrest. In the present study, GFAP at 12 h displayed a predictive value similar to that previously reported for NSE 48 h after OHCA, suggesting that GFAP at 12 h may be as good a neuroprognostic marker as NSE 36 h later [25–27]. However, interpretations are limited as NSE was used in patient care, impacting decisions on WLST and patient outcome. Additionally, NSE was analysed with an assay with 4% between-run imprecision, whereas the Simoa has been reported to have a larger variability (14%) [25, 28].

For tau, we found the highest predictive ability 48 h after OHCA, and two previous studies have reported similar predictive values after OHCA [4, 5]. In one of the studies, tau outperformed NSE at 48 h and 24 h in a select cohort of OHCA, a finding not replicated in the current study in a broader population of OHCA [5]. Both studies employed the same laboratory analysis methods for tau and NSE but differed in how haemolysis was handled in reporting NSE values. The same study reported a high predictive value of tau at 24 h after cardiac arrest [5]. This, in combination with our results, suggests that the earliest time point of clinically useful predictive value may be from around 24 h after cardiac arrest. We noted a temporal pattern in plasma tau levels where all patients, irrespective of outcome, appear to have an initial release with elevated tau, with decreasing levels in patients with good outcomes but a second burst in patients with poor outcomes. We speculate that the initial burst may reflect the opening of the blood–brain barrier or simply the release of tau from the peripheral nervous system directly into the bloodstream. This release pattern and association with outcome have been described previously in smaller studies [4, 6, 29]. Despite this pattern, employing multiple time points for outcome prediction was not superior to the last individual sample.

The present study included unselected OHCA and IHCA patients, rarely studied in post-cardiac arrest care. We found that prognostic abilities for all investigated biomarkers were generally lower after IHCA than OHCA, a finding also reported for NFL [22]. This is an important observation since evidence from OHCA is often extrapolated to inform the care of IHCA. Biomarkers of brain injury will fail to identify patients with poor outcome after cardiac arrest without significant post-hypoxic brain injury (e.g., haemodynamic collapse), possibly explaining the lower predictive value after IHCA. Withdrawal of life-support was more common after OHCA than IHCA. After OHCA WLST due to a poor neurological prognosis was more common whereas after IHCA WLST due to co-morbidity was more common, suggesting differences in degree of brain injury. Additionally, the number of patients in the IHCA group was small.

The results of the present study may be another step towards earlier and safe multimodal neuroprognostication. Biomarkers of brain injury in the blood may have an advantage early during post-cardiac arrest care when sedative agents are frequently administered and confound clinical examination and electroencephalography. We recently showed that NFL offers excellent outcome prediction as early as 12 h after OHCA in the same cohort used in the present study [22]. Additionally, the novel brain injury biomarker p-tau181 has been studied 24 h after OHCA and showed excellent outcome prediction but remains unstudied at earlier time points [10]. It may be that some biomarkers of brain injury, including GFAP and NFL, can provide useful neuroprognostic information even in patients affected by sedation 12 h after cardiac arrest. However, our studies on GFAP and NFL at 12 h after cardiac arrest were performed in the same patients and require validation. A combination of biomarkers may further improve neuroprognostication if the biomarker has different cellular origins. In the present study, the different combinations of GFAP, tau and NFL did not significantly improve prognostic performance compared to the most predictive biomarker alone. However, multiple biomarkers all suggest a similar outcome, improving the clinical safety of prognostication and reducing the risk of confounders or laboratory errors affecting decisions on WLST. Implementing novel biomarkers with 24/7 availability in clinical practice requires commercial assays, larger and more diverse studies to establish and validate outcome prediction cut-offs, and integrating the new biomarkers into routine clinical workflows. Collaborative efforts between researchers, healthcare institutions, and commercial partners are necessary to accelerate the translation of these biomarkers from research to clinical practice and ultimately improve the prediction of patient outcomes after cardiac arrest. Available commercial assays are Abbott Alinity (GFAP), Lumipulse (NFL) and Roche Elecsys (NFL, GFAP, tau). Still, they all require further validation of cut-offs for use in neuroprognostication after cardiac arrest.

### Strengths and limitations

This study is strengthened by its multicentre design, large sample size, prospective and standardised blood sampling and handling, comparison of IHCA and OHCA and batch analysis of biomarkers. Study limitations include the retrospective study design, where the data quality depends on the quality of medical records, the smaller sample size of IHCA, particularly at 48 h and the rate of WLST. Another limitation, particularly in the IHCA subgroup, is that the analyses include patients in which neuroprognostication may not be necessary due to awakening and those who die from other reasons than hypoxic brain injury. Due to the consent process, there may be missing data among survivors (but not non-survivors), which may bias the results towards patients with poor prognoses.

## Conclusion

GFAP and tau are promising novel biomarkers for predicting neurological outcome after cardiac arrest, with the highest predictive performance at 48 h after OHCA. The predictive ability of GFAP may be sufficiently high for clinical use at 12 h after cardiac arrest. NFL had similar or superior predictive performance at all time points.

### Supplementary Information

**Additional file 1.** Supplementary material including additional tables and figures.

## Data Availability

The protocol and the datasets analysed during the current study are available from the corresponding author upon reasonable request.

## Abbreviations

AUC: Area under the receiver operation curve
CI: Confidence interval
CPC: Cerebral performance category
CPR: Cardiopulmonary resuscitation
ERC: European Resuscitation Council
ESICM: European Society of Intensive Care Medicine
GCS-M: Glasgow Coma Scale-Motor Score
GFAP: Glial fibrillary acidic protein
ICU: Intensive care unit
IHCA: In-hospital cardiac arrest
IQR: Interquartile range
NFL: Neurofilament light
NSE: Neuron-specific enolase
OHCA: Out-of-hospital cardiac arrest
PCI: Percutaneous cardiac intervention
PEA: Pulseless electric activity
ROSC: Return of spontaneous circulation
TTM: Targeted temperature management
WLST: Withdrawal of life-sustaining therapy
