# Supplementary material for: Plasma glial fibrillary acidic protein and tau: predictors of neurological outcome after cardiac arrest
Source: Crit Care. 2024 Apr 9;28:116. doi: 10.1186/s13054-024-04889-0 (PMC11003115; doi:10.1186/s13054-024-04889-0)
Supplement: Supplementary file 1 — Additional file 1. Supplementary material including additional tables and figures. [file 13054_2024_4889_MOESM1_ESM.docx]

**Supplementary Material**

**Plasma glial fibrillary acidic protein and tau: Predictors of neurological outcome after cardiac arrest**

Isabelle Arctaedius^1*^, Helena Levin^2*^, Bergthora Thorgeirsdottir^3^ Marion Moseby-Knappe^4^, Tobias Cronberg^5^, Martin Annborn^6^, Niklas Nielsen^6^, Henrik Zetterberg^7-12^ Kaj Blennow^7-8^, Nicholas J. Ashton^8,13-15^, Attila Frigyesi^1^, Hans Friberg^3^, Anna Lybeck^1 #^ and Niklas Mattsson-Carlgren^5,16-17#^

*, # These authors contributed equally

1. Lund University, Skane University Hospital, Department of Clinical Sciences, Anaesthesia & Intensive Care, Lund, Sweden
2. Lund University, Department of Clinical Sciences, Anaesthesia & Intensive Care and Skane University Hospital, Department of Research & Education, Lund, Sweden
3. Lund University, Skane University Hospital, Department of Clinical Sciences, Anaesthesia & Intensive Care, Malmö, Sweden
4. Neurology and Rehabilitation Medicine, Department of Clinical Sciences Lund, Lund University, Skane University Hospital, Lund, Sweden.
5. Lund University, Skane University Hospital, Department of Clinical Sciences, Neurology, Lund, Sweden
6. Lund University, Helsingborg Hospital, Department of Clinical Sciences, Anaesthesia & Intensive Care, Helsingborg, Sweden
7. Clinical Neurochemistry Laboratory, Sahlgrenska University Hospital, Mölndal, Sweden.
8. Department of Psychiatry and Neurochemistry, Institute of Neuroscience and Physiology, The Sahlgrenska Academy at the University of Gothenburg, Mölndal, Sweden.
9. Department of Neurodegenerative Disease, UCL Institute of Neurology, Queen Square, London, UK
10. UK Dementia Research Institute at UCL, London, UK
11. Hong Kong Centre for Neurodegenerative Diseases, Clear Water Bay, Hong Kong, China
12. Wisconsin Alzheimer’s Disease Research Centre, University of Wisconsin School of Medicine and Public Health, University of Wisconsin-Madison, Madison, WI, USA
13. Institute of Psychiatry, Psychology and Neuroscience, King's College London, London, UK.
14. NIHR Biomedical Research Centre for Mental Health and Biomedical Research Unit for Dementia at South London and Maudsley NHS Foundation, London, UK.
15. Centre for Age-Related Medicine, Stavanger University Hospital, Stavanger, Norway.
16. Clinical Memory Research Unit, Department of Clinical Sciences, Lund University, Malmö, Sweden.
17. Wallenberg Centre for Molecular Medicine, Lund University, Lund, Sweden.

^*^ Corresponding author:

Isabelle Arctaedius (isabelle.arctaedius@med.lu.se)

Telephone: +46 (0)46 17 42 33

Department of Anaesthesia & Intensive Care

Skåne University Hospital

Entrégatan 7

SE-222 42 Lund Sweden

Orchid-ID: 0000-0001-7857-7393

**Table of Contents**

eTable 1: Neuroprognostic Markers and WLST……………………………………………………………………………………**3**

eTable 2: Performance of GFAP and tau after exclusion of patients who obeyed commands on admission……………………………………………………………………………………………………………………………………………**4**

eTable 3: Performance of GFAP and tau in patients with cardiac vs non-cardiac cause of arrest………..**5**

eFigure 1: GFAP and tau levels in patients with cardiac and non-cardiac cause of arrest……………………**6**

eTable 4: Cut-off levels for GFAP at high specificity for prediction of poor outcome…………..………………**7**

eTable 5: Cut-off levels for GFAP at high sensitivity for prediction of good outcome……………………….…**8**

eTable 6: Cut-off levels for tau at high specificity for prediction of poor outcome…………………….……….**9**

eTable 7: Cut-off levels for tau at high sensitivity for prediction of good outcome………………………..…**10**

eTable 8: Performance of GFAP using combined measurements **11**

eTable 9: Performance of total tau using combined measurements **12**

eTable 10: Prognostic performance of GFAP and total tau**13**

eTable 11: Prognostic performance of GFAP vs NSE**14**

eTable 12: Prognostic performance of total tau vs NSE**15**

eTable 13: Prognostic performance of GFAP and NFL**16**

eTable 14: Prognostic performance of total tau and NFL**17**

**eTable 1. Neuroprognostic markers and WLST**

|  | OHCA n=328 | IHCA n=100 |
| --- | --- | --- |
| Neuroprognostic markers n=pathological/available (%) |  |  |
| GCS-M 1-3 ≥72hrs ^a^ | 122/170 (71.8) | 20/30 (66.7) |
| Pupillary light reflexes bilaterally absent ≥72hrs ^a^ | 38/155 (24.5) | 8/27 (29.6) |
| Corneal reflexes bilaterally absent ≥72hrs ^a^ | 52/141 (36.9) | 11/22 (50) |
| Status myoclonus 0-72h | 32/328 (9.8) | 5/100 (5) |
| NSE 48h >60ug/L | 109/216 (50.5) | 15/51 (29.4) |
| NSE 72h >60ug/L | 63/140 (45) | 13/37 (35.1) |
| SSEP N20 bilaterally absent | 62/110 (56.4) | 14/23 (60.9) |
| EEG highly malignant with or without ongoing sedation ≥48hrs | 68/165 (41.2) | 15/31 (48.4) |
| EEG highly malignant without ongoing sedation ≥48hrs | 66/158 (41.8) | 15/30 (50) |
| CT: diffuse and extensive anoxic injury | 74/258 (28.7) | 7/48 (14.6) |
| MRI: diffuse and extensive anoxic injury | 31/46 (67.4) | 6/12 (50) |
| ≥2 markers of poor neurological prognosis according to ERC/ESCIM algorithm ^b^ | 119/328 (36.3) | 20/100 (20) |
| Reason for WLST^c^ |  |  |
| WLST | 181 (55.2) | 34 (34) |
| Days from CA to WLST | 4 (2-5)^e^ | 4 (3-6)^d^ |
| WLST due to poor neurological prognosis, n (%) | 159 (89.8) | 26 (81.3) |
| WLST solely due to poor neurological prognosis | 105 (59.3) | 18 (56.3) |
| WLST due to multi-organ failure or failing circulation, n (%) | 27 (15.3) | 4 (12.5) |
| WLST due to medical co-morbidity, n (%) | 13 (7.3) | 8 (25) |
| WLST due to ethical reasons, n (%) | 41 (23.2) | 4 (12.5) |

Continuous variables presented as median (IQR) and categorical variables as n(%).

^a^ Recorded at formal neuroprognostication recommended at ≥72 hours post-cardiac arrest and here as with or without ongoing sedation.
^b^ Nolan JP et al. European Resuscitation Council and European Society of Intensive Care Medicine Guidelines 2021: Post-resuscitation care. Resuscitation. 2021;161:220-69 (2).
^c^ There can be more than one reason for WLST.
Missing data: ^d^ n=2  ^e^ n=4.

CT: computed tomography; EEG: electroencephalogram; MRI: magnetic resonance imaging; NSE: serum neuron-specific enolase; SSEP: short-latency somatosensory evoked potentials; S. myoclonus: generalized status myoclonus; WLST: withdrawal of life-sustaining therapy

| **Biomarker** | **Location of arrest** | **Time point** | **GCSM 1-6** | | **GSC-M 1-5** | | **p-value** |
| --- | --- | --- | --- | --- | --- | --- | --- |
|  |  |  | **N** | **AUC (95% CI)** | **N** | **AUC (95% CI)** |  |
| GFAP | OHCA | 0 h | 289 | 0.76 (0.70-0.82) | 275 | 0.74 (0.68-0.80) | 0.98 |
|  |  | 12 h | 300 | 0.86 (0.81-0.90) | 284 | 0.85 (0.80-0.90) | 0.98 |
|  |  | 48 h | 210 | 0.91 (0.87-0.96) | 201 | 0.90 (0.86-0.95) | 0.98 |
|  | IHCA | 0 h | 83 | 0.77 (0.66-0.87) | 63 | 0.74 (0.61-0.86) | 0.98 |
|  |  | 12 h | 87 | 0.83 (0.74-0.92) | 66 | 0.82 (0.72-0.92) | 0.98 |
|  |  | 48 h | 54 | 0.83 (0.71-0.95) | 45 | 0.83 (0.69-0.97) | 0.98 |
| tau | OHCA | 0 h | 289 | 0.72 (0.66-0.79) | 275 | 0.71 (0.65-0.78) | 0.99 |
|  |  | 12 h | 300 | 0.75 (0.69-0.81) | 284 | 0.75 (0.69-0.81) | 0.99 |
|  |  | 48 h | 210 | 0.93 (0.89-0.96) | 201 | 0.93 (0.90-0.96) | 0.99 |
|  | IHCA | 0 h | 83 | 0.61 (0.49-0.74) | 63 | 0.57 (0.41-0.72) | 0.99 |
|  |  | 12 h | 87 | 0.68 (0.56-0.79) | 66 | 0.65 (0.51-0.79) | 0.99 |
|  |  | 48 h | 54 | 0.77 (0.65-0.90) | 45 | 0.79 (0.66-0.92) | 0.99 |

**eTable 2.** **Prognostic performance of GFAP and tau after exclusion of patients who obeyed commands (GCS-M 6) on admission**

Prognostic performance of GFAP and tau assessed by the area under the receiver-operating curve (AUC), defined from logistic regression models in all included patients and a sub-cohort where patients with Glascow Come Scale Motor (GSC-M) score 6 were excluded. P-values for group comparisons were calculated with the DeLong method and adjusted for multiplicity (n=6) using FDR correction. *GFAP* glial fibrillary protein, *CI* Confidence Interval, *OHCA* out-of-hospital cardiac arrest, *IHCA* in-hospital cardiac arrest

**eTable 3. Performance of GFAP and tau in patients with cardiac vs non-cardiac cause of arrest**

| **Biomarker** | **Location of arrest** | **Time point** | **Cardiac cause** | | **Non-cardiac cause** | | **p-value** |
| --- | --- | --- | --- | --- | --- | --- | --- |
|  |  |  | **N (% CPC3-5)** | **AUC (95% CI)** | **N (% CPC3-5)** | **AUC (95% CI)** |  |
| GFAP | OHCA | 0 h | 217 (62) | 0.77 (0.71-0.84) | 72 (92) | 0.85 (0.71-0.98) | 0.71 |
|  |  | 12 h | 226 (61) | 0.86 (0.81-0.91) | 74 (91) | 0.91 (0.83-0.98) | 0.71 |
|  |  | 48 h | 158 (61) | 0.91 (0.85-0.96) | 52 (90) | 0.96 (0.91-1.00) | 0.71 |
|  | IHCA | 0 h | 31 (36) | 0.76 (0.58-0.94) | 52 (65) | 0.81 (0.68-0.94) | 0.75 |
|  |  | 12 h | 30 (40) | 0.80 (0.63-0.98) | 57 (65) | 0.85 (0.75-0.96) | 0.75 |
|  |  | 48 h | 20 (45) | 0.89 (0.72-1.00) | 34 (77) | 0.86 (0.71-1.00) | 0.79 |
| tau | OHCA | 0 h | 217 (62) | 0.71 (0.64-0.79) | 72 (92) | 0.86 (0.72-1.00) | 0.24 |
|  |  | 12 h | 226 (61) | 0.76 (0.69-0.82) | 74 (91) | 0.84 (0.71-0.98) | 0.39 |
|  |  | 48 h | 158 (61) | 0.94 (0.91-0.98) | 52 (90) | 0.88 (0.78-0.97) | 0.39 |
|  | IHCA | 0 h | 31 (36) | 0.47 (0.24-0.69) | 52 (65) | 0.75 (0.61-0.90) | 0.19 |
|  |  | 12 h | 30 (40) | 0.63 (0.42-0.85) | 57 (65) | 0.75 (0.62-0.88) | 0.43 |
|  |  | 48 h | 20 (45) | 0.82 (0.63-1.00) | 34 (77) | 0.76 (0.59-0.92) | 0.64 |

Prognostic performance of GFAP and tau in patients with cardiac vs non-cardiac cause of arrest. The area under the receiver-operating curve (AUC) was defined from logistic regression models. P-values for group comparisons were calculated with the DeLong method and adjusted for multiplicity (n=6) using FDR correction. *GFAP* glial fibrillary protein, *CI* Confidence Interval, *OHCA* out-of-hospital cardiac arrest, *IHCA* in-hospital cardiac arrest

**eFigure 1. GFAP and tau levels in patients with cardiac and non-cardiac cause of arrest**


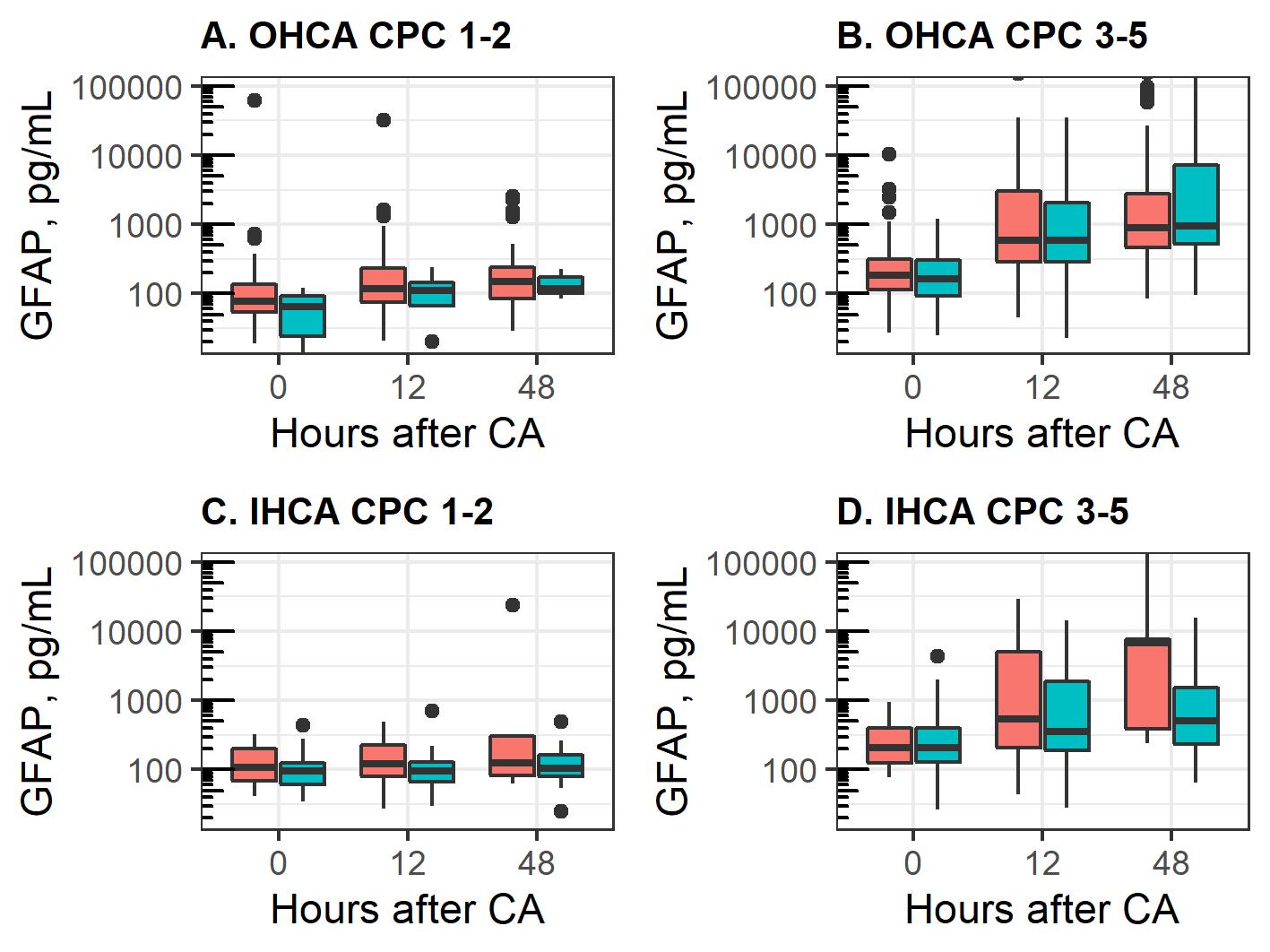

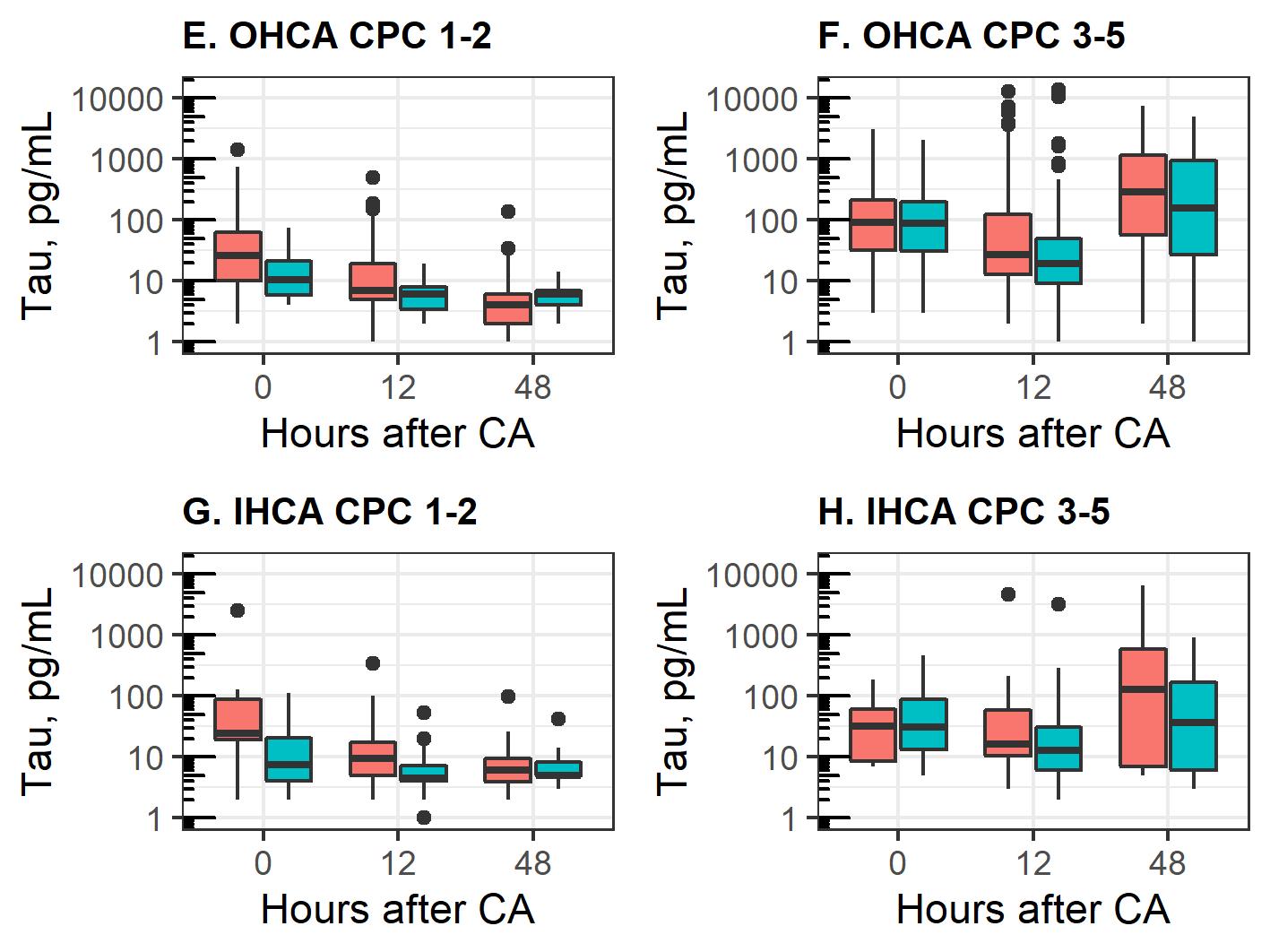

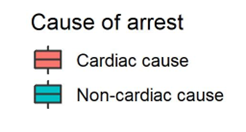


**GFAP**

Boxplots of GFAP (A-D) and tau levels (E-H) in patients with cardiac and non-cardiac cause of arrest. The boxes show the median and interquartile range of GFAP and tau in patients with good (CPC 1-2) and poor (CPC 3-5) outcomes in OHCA (A,E and B,F at 0, 12, 48h; 89, 96, 66 and 200, 204, 144 samples) and in IHCA patients (C,G and D,H at 0, 12, 48h; 38, 38, 19 and 45, 49, 35 samples). *OHCA* out-of-hospital cardiac arrest, *IHCA* in-hospital cardiac arrest, *CPC* Cerebral Performance Category

**eTable 4. Cut-off levels for GFAP at high specificity for prediction of poor outcome**

|  | **Time** | **FPR %** | **GFAP cut-off (pg/ml)** | **Specificity (95% CI)** | **Sensitivity (95% CI)** | **True Positive** | **False Positive** | **True Negative** | **False Negative** | **Total** |
| --- | --- | --- | --- | --- | --- | --- | --- | --- | --- | --- |
| OHCA | 12h | ≤5 | 1330 | 0.96 (0.92-0.99) | 0.37(0.30-0.44) | 75 | 4 | 92 | 129 | 300 |
|  |  | ≤2 | 1626 | 0.99 (0.97-1) | 0.34 (0.27-0.41) | 69 | 1 | 95 | 135 | 300 |
|  |  | 0 | 33465 | 1 (1-1) | 0.03 (0.01-0.06) | 7 | 0 | 96 | 197 | 300 |
|  | 48h | ≤5 | 1534 | 0.95 (0.89-1) | 0.40 (0.31-0.48) | 57 | 3 | 63 | 87 | 210 |
|  |  | ≤2 | 2376 | 0.98 (0.95-1) | 0.32 (0.24-0.40) | 46 | 1 | 65 | 98 | 210 |
|  |  | 0 | 2591 | 1 (1-1) | 0.31 (0.24-0.39) | 45 | 0 | 66 | 99 | 210 |
| IHCA | 12h | ≤5 | 562 | 0.97 (0.92-1) | 0.43 (0.29-0.57) | 21 | 1 | 37 | 28 | 87 |
|  |  | ≤2* | - | - | - | - | - | - | - | - |
|  |  | 0 | 727.5 | 1 (1-1) | 0.37 (0.24-0.51) | 18 | 0 | 38 | 31 | 87 |
|  | 48h | ≤5* | - | - | - | - | - | - | - | - |
|  |  | ≤2* | - | - | - | - | - | - | - | - |
|  |  | 0 | 95628.5 | 1 (1-1) | 0.03 (0-0.09) | 1 | 0 | 19 | 34 | 54 |

GFAP cut-off values achieving a FPR of 0%, ≤2%, and ≤5% for prediction of poor outcome at 12­ and 48 hours post-cardiac arrest. A bootstrap method (10000 iterations) was used to calculate 95% confidence intervals for sensitivity and specificity. * Cut-off values were not available. *FPR* false positive rate, *CI* confidence interval, *GFAP* Glial fibrillary acidic protein

**eTable 5. Cut-off levels for GFAP at high sensitivity for prediction of good outcome**

|  | **Time** | **FNR %** | **GFAP cut-off (pg/ml)** | **Specificity** | **Sensitivity (95% CI)** | **NPV** | **True Positive** | **False Positive** | **True Negative** | **False Negative** | **Total** |
| --- | --- | --- | --- | --- | --- | --- | --- | --- | --- | --- | --- |
| OHCA | 12h | ≤20 | 244.5 | 0.79 (0.71-0.88) | 0.80 | 0.66 | 164 | 20 | 76 | 40 | 300 |
|  |  | ≤15 | 191.5 | 0.70 (0.60-0.79) | 0.85 | 0.69 | 174 | 29 | 67 | 30 | 300 |
|  |  | ≤10 | 150 | 0.60 (0.50-0.70) | 0.90 | 0.74 | 184 | 38 | 58 | 20 | 300 |
|  |  | ≤5 | 110.5 | 0.47 (0.38-0.56) | 0.95 | 0.82 | 194 | 51 | 45 | 10 | 300 |
|  | 48h | ≤20 | 418 | 0.91 (0.83-0.97) | 0.81 | 0.68 | 116 | 6 | 60 | 28 | 210 |
|  |  | ≤15 | 353.5 | 0.89 (0.82-0.95) | 0.85 | 0.74 | 123 | 7 | 59 | 21 | 210 |
|  |  | ≤10 | 253.5 | 0.80 (0.70-0.89) | 0.90 | 0.79 | 130 | 13 | 53 | 14 | 210 |
|  |  | ≤5 | 183 | 0.64 (0.52-0.76) | 0.95 | 0.86 | 137 | 24 | 42 | 7 | 210 |
| IHCA | 12h | ≤20 | 166 | 0.71 (0.55-0.84) | 0.82 | 0.75 | 40 | 11 | 27 | 9 | 87 |
|  |  | ≤15 | 145.5 | 0.68 (0.53-0.82) | 0.86 | 0.79 | 42 | 12 | 26 | 7 | 87 |
|  |  | ≤10 | 80.5 | 0.37 (0.21-0.53) | 0.92 | 0.78 | 45 | 24 | 14 | 4 | 87 |
|  |  | ≤5 | 50.5 | 0.21 (0.08-0.34) | 0.96 | 0.80 | 47 | 30 | 8 | 2 | 87 |
|  | 48h | ≤20 | 236 | 0.68 (0.47-0.89) | 0.80 | 0.65 | 28 | 6 | 13 | 7 | 54 |
|  |  | ≤15 | 182 | 0.63 (0.42-0.84) | 0.86 | 0.71 | 30 | 7 | 12 | 5 | 54 |
|  |  | ≤10 | 112 | 0.53 (0.32-0.74) | 0.91 | 0.77 | 32 | 9 | 10 | 3 | 54 |
|  |  | ≤5 | 93 | 0.37 (0.16-0.58) | 0.97 | 0.88 | 34 | 12 | 7 | 1 | 54 |

Cut-off values for GFAP achieving a FNR of 20, 15, 10, 5% for prediction of good outcome at 12 and 48 hours post-cardiac arrest. A bootstrap method (10000 iterations) was used to calculate 95% confidence intervals for sensitivity and specificity. *FNR* false negative rate, *NPV* negative predictive value, *CI* confidence interval

**eTable 6. Cut-off levels for tau at high specificity for prediction of poor outcome**

|  | **Time** | **FPR %** | **Tau cut-off (pg/ml)** | **Specificity (95% CI)** | **Sensitivity (95% CI)** | **True Positive** | **False Positive** | **True Negative** | **False Negative** | **Total** |
| --- | --- | --- | --- | --- | --- | --- | --- | --- | --- | --- |
| OHCA | 12h | ≤5 | 146 | 0.96 (0.92-0.99) | 0.22 (0.17-0.28) | 45 | 4 | 92 | 159 | 300 |
|  |  | ≤2 | 198.5 | 0.99 (0.97-1) | 0.22(0.17-0.28) | 45 | 1 | 95 | 159 | 300 |
|  |  | 0 | 502.5 | 1 (1-1) | 0.15 (0.10-0.20) | 30 | 0 | 96 | 174 | 300 |
|  | 48h | ≤5 | 27.5 | 0.95 (0.89-1) | 0.79 (0.72-0.85) | 114 | 3 | 63 | 30 | 210 |
|  |  | ≤2 | 35.5 | 0.98 (0.95-1) | 0.76 (0.69-0.83) | 109 | 1 | 65 | 35 | 210 |
|  |  | 0 | 140.5 | 1 (1-1) | 0.58 (0.5-0.67) | 84 | 0 | 66 | 60 | 210 |
| IHCA | 12h | ≤5 | 157 | 0.97 (0.92-1) | 0.08 (0.02-0.16) | 4 | 1 | 37 | 45 | 87 |
|  |  | ≤2* | - | - | - | - | - | - | - | - |
|  |  | 0 | 1756.5 | 1 (1-1) | 0.04 (0-0.10) | 2 | 0 | 38 | 47 | 87 |
|  | 48h | ≤5* | - | - | - | - | - | - | - | - |
|  |  | ≤2* | - | - | - | - | - | - | - | - |
|  |  | 0 | 102.5 | 1 (1-1) | 0.43 (0.26-0.6) | 15 | 0 | 19 | 20 | 54 |

Tau cut-off values achieving a FPR of 0%, 2%, and 5% for prediction of poor outcome at 12 and 48 hours post-cardiac arrest. A bootstrap method (10000 iterations) was used to calculate 95% confidence intervals for sensitivity and specificity. * Cut-off values were not available. *FPR* false positive rate, *CI* confidence interval

**eTable 7 . Cut-off levels for tau at high sensitivity for prediction of good outcome**

|  | **Time** | **FNR %** | **Tau cut-off (pg/ml)** | **Specificity (95% CI)** | **Sensitivity (95% CI)** | **NPV**  **%** | **True Positive** | **False Positive** | **True Negative** | **False Negative** | **Total** |
| --- | --- | --- | --- | --- | --- | --- | --- | --- | --- | --- | --- |
| OHCA | 12h | ≤20 | 8.5 | 0.56 (0.46-0.67) | 0.81 | 0.59 | 42 | 54 | 38 | 166 | 300 |
|  |  | ≤15 | 6.5 | 0.42 (0.32-0.52) | 0.88 | 0.63 | 56 | 40 | 24 | 180 | 300 |
|  |  | ≤10 | 5.5 | 0.29 (0.20-0.39) | 0.93 | 0.65 | 68 | 28 | 15 | 189 | 300 |
|  |  | ≤5 | 4.5 | 0.22 (0.14-0.30) | 0.95 | 0.68 | 75 | 21 | 10 | 194 | 300 |
|  | 48h | ≤20 | 24.5 | 0.94 (0.88-0.98) | 0.81 | 0.69 | 4 | 62 | 28 | 116 | 210 |
|  |  | ≤15 | 12.5 | 0.82 (0.73-0.91) | 0.85 | 0.72 | 12 | 54 | 21 | 123 | 210 |
|  |  | ≤10 | 5.5 | 0.68 (0.58-0.79) | 0.92 | 0.80 | 21 | 45 | 12 | 132 | 210 |
|  |  | ≤5 | 3.5 | 0.45 (0.33-0.58) | 0.96 | 0.83 | 36 | 30 | 6 | 138 | 210 |
| IHCA | 12h | ≤20 | 4.5 | 0.34 (0.18-0.50) | 0.84 | 0.62 | 41 | 25 | 13 | 8 | 87 |
|  |  | ≤15* | - | - | - | - | - | - | - | - | - |
|  |  | ≤10 | 3.5 | 0.13 (0.03-0.24) | 0.92 | 0.56 | 45 | 33 | 5 | 4 | 87 |
|  |  | ≤5 | 2.5 | 0.11(0.03-0.21) | 0.98 | 0.80 | 48 | 34 | 4 | 1 | 87 |
|  | 48h | ≤20 | 5.5 | 0.53 (0.32-0.74) | 0.83 | 0.63 | 29 | 9 | 10 | 6 | 54 |
|  |  | ≤15 | 4.5 | 0.21 (0.05-0.42) | 0.89 | 0.50 | 31 | 15 | 4 | 4 | 54 |
|  |  | ≤10 | 3.5 | 0.21 (0.05-0.42) | 0.94 | 0.67 | 33 | 15 | 4 | 2 | 54 |
|  |  | ≤5* | - | - | - | - | - | - | - | - | - |

Cut-off values for tau levels achieving a FNR of 20, 15, 10, 5% for prediction of good outcome at 12 and 48 hours post-cardiac arrest. A bootstrap method (10000 iterations) was used to calculate 95% confidence intervals for sensitivity and specificity. * Cut-off values were not available. *FNR* false negative rate, *NPV* negative predictive value, *CI* confidence interval

**eTable 8. Performance of GFAP using combined measurements**

|  | **0h** | **12h** | **48h** | **N** | **AUC (95% CI)**  **Last time point** | **AUC (95% CI)**  **Last time point**  **+ Δ Difference** | **p-value** |
| --- | --- | --- | --- | --- | --- | --- | --- |
| OHCA | Yes | Yes |  | 269 | 0.87 (0.82-0.92) | 0.87 (0.82-0.91) | 1.00 |
|  | Yes |  | Yes | 186 | 0.92 (0.87-0.96) | 0.92 (0.88-0.97) | 0.60 |
|  |  | Yes | Yes | 191 | 0.90 (0.85-0.95) | 0.91 (0.86-0.96) | 0.60 |
|  | Yes | Yes | Yes | 173 | 0.91 (0.86-0.96) | 0.92 (0.88-0.97) | 0.60 |
| IHCA | Yes | Yes |  | 72 | 0.81 (0.71-0.91) | 0.81 (0.71-0.91) | 0.67 |
|  | Yes |  | Yes | 47 | 0.83 (0.70-0.96) | 0.82 (0.69-0.95) | 0.67 |
|  |  | Yes | Yes | 42 | 0.77 (0.60-0.94) | 0.86 (0.75-0.97) | 0.60 |
|  | Yes | Yes | Yes | 36 | 0.78 (0.60-0.96) | 0.84 (0.70-0.97) | 0.67 |

Prognostic performance of combined vs single measurements assessed by the area under the receiver-operating curve (AUC), defined from logistic regression models. The DeLong method was used to test if the Δ change in GFAP levels between two time points increased the prognostic performance compared to the using GFAP levels of only the last time point in the model. Each model included samples from the time points, admission (0h), 12h, 48h, as shown in the table. P-values were adjusted for multiplicity (n=8) using FDR correction. *GFAP* glial fibrillary protein, *CI* Confidence Interval, *OHCA* out-of-hospital cardiac arrest, *IHCA* in-hospital cardiac arrest, *FDR* false discovery rate

**eTable 9. Performance of total tau using combined measurements**

|  | **0h** | **12h** | **48h** | **N** | **AUC (95% CI)**  **Last time point** | **AUC (95% CI)**  **Last time point**  **+ Δ Difference** | **p-value** |
| --- | --- | --- | --- | --- | --- | --- | --- |
| OHCA | Yes | Yes |  | 269 | 0.75 (0.69-0.81) | 0.76 (0.70-0.82) | 0.82 |
|  | Yes |  | Yes | 186 | 0.92 (0.88-0.96) | 0.92 (0.88-0.95) | 0.69 |
|  |  | Yes | Yes | 191 | 0.92 (0.88-0.95) | 0.92 (0.88-0.96) | 0.94 |
|  | Yes | Yes | Yes | 173 | 0.91 (0.87-0.95) | 0.93 (0.89-0.97) | 0.40 |
| IHCA | Yes | Yes |  | 72 | 0.67 (0.54-0.80) | 0.67 (0.54-0.80) | 0.94 |
|  | Yes |  | Yes | 47 | 0.75 (0.62-0.89) | 0.76 (0.63-0.90) | 0.94 |
|  |  | Yes | Yes | 42 | 0.77 (0.62-0.92) | 0.76 (0.62-0.91) | 0.94 |
|  | Yes | Yes | Yes | 36 | 0.77 (0.61-0.92) | 0.73 (0.57-0.90) | 0.94 |

Prognostic performance of combined vs single measurements assessed by the area under the receiver-operating curve (AUC), defined from logistic regression models. The DeLong method was used to test if the Δ change in tau levels between two time points increased the prognostic performance compared to the using tau levels of only the last time point in the model. Each model included samples from the time points, admission (0h), 12h, 48h, as shown in the table. P-values were adjusted for multiplicity (n=8) using FDR correction. *CI* Confidence Interval, *OHCA* out-of-hospital cardiac arrest, *IHCA* in-hospital cardiac arrest, *FDR* false discovery rate

**eTable 10. Prognostic performance of GFAP and total tau**

|  | **Time point** | **N** | **AUC (95% CI)**  **GFAP** | **AUC (95% CI)**  **tau** | **AUC (95% CI)**  **GFAP + tau** | **p-value**  **GFAP**  **vs tau** | **p-value**  **GFAP + tau**  **vs. GFAP** | **p-value**  **GFAP + tau**  **vs. tau** |
| --- | --- | --- | --- | --- | --- | --- | --- | --- |
| OHCA | 0 h | 289 | 0.76 (0.70-0.82) | 0.72 (0.66-0.79) | 0.78 (0.73-0.84) | 0.46 | 0.56 | 0.02 |
|  | 12 h | 300 | 0.86 (0.81-0.90) | 0.75 (0.69-0.81) | 0.86 (0.81-0.90) | 0.003 | 0.60 | <0.001 |
|  | 48 h | 210 | 0.91 (0.87-0.96) | 0.93 (0.89-0.96) | 0.95 (0.92-0.98) | 0.51 | 0.11 | 0.05 |
| IHCA | 0 h | 83 | 0.77 (0.66-0.87) | 0.61 (0.49-0.74) | 0.76 (0.66-0.86) | 0.08 | 0.63 | 0.04 |
|  | 12 h | 87 | 0.83 (0.74-0.92) | 0.68 (0.56-0.79) | 0.83 (0.74-0.91) | 0.04 | 0.63 | 0.03 |
|  | 48 h | 54 | 0.83 (0.71-0.95) | 0.77 (0.65-0.90) | 0.86 (0.74-0.98) | 0.46 | 0.56 | 0.09 |

Predictive performance of glial fibrillary protein (GFAP) combined with tau on admission (0 h), at 12 h, and at 48 h after OHCA and IHCA. P-values for comparisons were calculated with the DeLong method and adjusted for multiplicity (n=6) using FDR correction. *AUC* area under the receiver operating characteristic curve, *CI* Confidence Interval, *OHCA* out-of-hospital cardiac arrest, *IHCA* in-hospital cardiac arrest, *FDR* false discovery rate

**eTable 11. Prognostic performance of GFAP vs NSE**

|  | **Biomarkers** | **N** | **AUC (95% CI)**  **GFAP** | **AUC (95% CI)**  **NSE** | **p-value**  **GFAP vs NSE** |
| --- | --- | --- | --- | --- | --- |
| OHCA | GFAP 12h vs NSE 24h | 232 | 0.86 (0.81-0.91) | 0.83 (0.77-0.88) | 0.42 |
|  | GFAP 12h vs NSE 48h | 201 | 0.83 (0.76-0.89) | 0.89 (0.85-0.94) | 0.08 |
|  | GFAP 48h vs NSE 48h | 190 | 0.91 (0.86-0.96) | 0.90 (0.86-0.94) | 0.86 |
| IHCA | GFAP 12h vs NSE 24h | 59 | 0.88 (0.79-0.97) | 0.71 (0.57-0.86) | 0.08 |
|  | GFAP 12h vs NSE 48h | 42 | 0.91 (0.83-1.00) | 0.72 (0.57-0.88) | 0.08 |
|  | GFAP 48h vs NSE 48h | 41 | 0.83 (0.68-0.98) | 0.77 (0.63-0.92) | 0.52 |

Prognostic performance of glial fibrillary protein (GFAP) and neuron-specific enolase (NSE) measured with the area under the curve (AUC) in a subgroup of OHCA and IHCA patients. P-values were calculated with the DeLong method and adjusted for multiplicity (n=6) using FDR correction. Samples were collected and analysed for NSE as part of the clinical routine at 24 and 48h after cardiac arrest. Only participants with both NSE and GFAP levels were included and therefore the results differ slightly from those in Figure 3 and 4. *CI* Confidence Interval, *OHCA* out-of-hospital cardiac arrest, *IHCA* in-hospital cardiac arrest, *FDR* false discovery rate

**eTable 12. Prognostic performance of tau vs NSE**

|  | **Biomarkers** | **N** | **AUC (95% CI)**  **tau** | **AUC (95% CI)**  **NSE** | **p-value**  **tau vs NSE** |
| --- | --- | --- | --- | --- | --- |
| OHCA | tau 12h vs NSE 24h | 232 | 0.71 (0.64-0.78) | 0.83 (0.77-0.88) | <0.001 |
|  | tau 12h vs NSE 48h | 201 | 0.67 (0.59-0.76) | 0.89 (0.85-0.94) | <0.001 |
|  | tau 48h vs NSE 48h | 190 | 0.92 (0.88-0.96) | 0.90 (0.86-0.94) | 0.59 |
| IHCA | tau 12h vs NSE 24h | 59 | 0.69 (0.54-0.83) | 0.71 (0.57-0.86) | 0.68 |
|  | tau 12h vs NSE 48h | 42 | 0.62 (0.43-0.81) | 0.72 (0.57-0.88) | 0.59 |
|  | tau 48h vs NSE 48h | 41 | 0.74 (0.59-0.89) | 0.77 (0.63-0.92) | 0.62 |

Prognostic performance of total-tau and neuron-specific enolase (NSE) measured with the area under the curve (AUC) in a subgroup of OHCA and IHCA patients. P-values were calculated with the DeLong method and adjusted for multiplicity (n=6) using FDR correction. Samples were collected and analysed for NSE as part of the clinical routine at 24 and 48h after cardiac arrest. Only participants with both NSE and GFAP levels were included and therefore the results differ slightly from those in Figure 3 and 4. *CI* Confidence Interval, *OHCA* out-of-hospital cardiac arrest, *IHCA* in-hospital cardiac arrest, *FDR* false discovery rate

**eTable 13. Prognostic performance of GFAP and NFL**

|  | **Time point** | **N** | **AUC (95% CI)**  **NFL** | **AUC (95% CI)**  **GFAP** | **AUC (95% CI)**  **GFAP + NFL** | **p-value GFAP vs NFL** | **p-value**  **GFAP + NFL vs. GFAP** | **p-value**  **GFAP + NFL**  **vs. NFL** |
| --- | --- | --- | --- | --- | --- | --- | --- | --- |
| OHCA | 0 h | 289 | 0.77 (0.71-0.83) | 0.76 (0.70-0.82) | 0.79 (0.73-0.84) | 0.78 | 0.36 | 0.36 |
|  | 12 h | 300 | 0.93 (0.90-0.96) | 0.86 (0.81-0.90) | 0.93 (0.91-0.96) | 0.01 | <0.001 | 0.59 |
|  | 48 h | 210 | 0.97 (0.95-0.99) | 0.91 (0.87-0.96) | 0.97 (0.96-0.99) | 0.02 | 0.005 | 0.59 |
| IHCA | 0 h | 83 | 0.67 (0.56-0.79) | 0.77 (0.66-0.87) | 0.78 (0.67-0.88) | 0.20 | 0.56 | 0.16 |
|  | 12 h | 87 | 0.81 (0.72-0.90) | 0.83 (0.74-0.92) | 0.85 (0.76-0.93) | 0.75 | 0.36 | 0.36 |
|  | 48 h | 54 | 0.86 (0.77-0.96) | 0.83 (0.71-0.95) | 0.87 (0.78-0.97) | 0.75 | 0.37 | 0.63 |

Predictive performance of glial fibrillary protein (GFAP) combined with neurofilament light (NFL) on admission (0 h), at 12 h, and at 48 h after OHCA and IHCA. P-values for comparisons were calculated with the DeLong method and adjusted for multiplicity (n=6) using FDR correction. *AUC* area under the receiver operating characteristic curve, *CI* Confidence Interval, *OHCA* out-of-hospital cardiac arrest, *IHCA* in-hospital cardiac arrest, *FDR* false discovery rate

**eTable 14. Prognostic performance of tau and NFL**

|  | **Time point** | **N** | **AUC (95% CI)**  **NFL** | **AUC (95% CI)**  **tau** | **AUC (95% CI)**  **tau + NFL** | **p-value**  **tau vs NFL** | **p-value**  **tau + NFL vs. tau** | **p-value**  **tau + NFL**  **vs. NFL** |
| --- | --- | --- | --- | --- | --- | --- | --- | --- |
| OHCA | 0 h | 289 | 0.77 (0.71-0.83) | 0.72 (0.66-0.79) | 0.80 (0.74-0.85) | 0.27 | 0.004 | 0.76 |
|  | 12 h | 300 | 0.93 (0.90-0.96) | 0.75 (0.69-0.81) | 0.93 (0.90-0.96) | <0.001 | <0.001 | 0.85 |
|  | 48 h | 210 | 0.97 (0.95-0.99) | 0.93 (0.89-0.96) | 0.97 (0.95-0.99) | 0.004 | <0.003 | 0.85 |
| IHCA | 0 h | 83 | 0.67 (0.56-0.79) | 0.61 (0.49-0.74) | 0.71 (0.60-0.82) | 0.45 | 0.08 | 0.76 |
|  | 12 h | 87 | 0.81 (0.72-0.90) | 0.68 (0.56-0.79) | 0.80 (0.71-0.90) | 0.08 | 0.04 | 0.85 |
|  | 48 h | 54 | 0.86 (0.77-0.96) | 0.77 (0.65-0.90) | 0.86 (0.77-0.96) | 0.22 | 0.08 | 0.85 |

Predictive performance of tau combined with neurofilament light (NFL) on admission (0 h), at 12 h, and at 48 h after OHCA and IHCA. P-values for comparisons were calculated with the DeLong method and adjusted for multiplicity (n=6) using FDR correction. *AUC* area under the receiver operating characteristic curve, *CI* Confidence Interval, *OHCA* out-of-hospital cardiac arrest, *IHCA* in-hospital cardiac arrest, *FDR* false discovery rate
